# Comparison of Metabolic and Morphological Response Criteria for Early Prediction of Response and Survival in NSCLC Patients Treated With Anti-PD-1/PD-L1

**DOI:** 10.3389/fonc.2020.01090

**Published:** 2020-07-31

**Authors:** Angelo Castello, Sabrina Rossi, Luca Toschi, Egesta Lopci

**Affiliations:** ^1^Department of Nuclear Medicine, Humanitas Clinical and Research Hospital—IRCCS, Milan, Italy; ^2^Medical Oncology, Humanitas Clinical and Research Hospital—IRCCS, Milan, Italy

**Keywords:** non-small cell lung cancer, checkpoint inhibitors, ^18^F-FDG PET/CT, RECIST, EORTC, PERCIST, imPERCIST, PERCIMT

## Abstract

**Introduction/Aim:** Immunotherapy with immune checkpoint inhibitors (ICIs) has positively changed the history of several malignant tumors. In parallel, new challenges have emerged in the evaluation of treatment response as a result of their peculiar anticancer effect. In the current study, we aimed to compare different response criteria, both morphological and metabolic, for assessing response and outcome in patients with advanced non-small cell lung cancer (NSCLC) treated with ICI.

**Materials and Methods:** Overall, 52 patients with advanced NSCLC candidate to ICI were prospectively evaluated. Inclusion criteria comprised whole-body contrast-enhanced CT and ^18^F-FDG PET/CT at baseline and at the first response evaluation 3 or 4 cycles after ICI. Response assessment on CT was performed according to RECIST 1.1 and imRECIST criteria, whereas metabolic response on PET was computed by EORTC, PERCIST, imPERCIST, and PERCIMT criteria. The concordance between the different tumor response criteria and the performance of each criterion to predict progression-free survival (PFS) and overall survival (OS) were calculated.

**Results:** Inclusion criteria were fulfilled in 35 out of 52 patients. We observed a low agreement between imRECIST and imPERCIST (κ = 0.143) with discordant response in 20 patients, particularly regarding stable disease and progressive disease groups. Fair agreement between imRECIST and EORTC (κ = 0.340), and PERCIST (κ = 0.342), and moderate for PERCIMT (κ = 0.413) were detected. All criteria were significantly associated with PFS, while only PERCIMT and imPERCIST were associated with OS. Of note, in patients classified as immune stable disease (iSD), imPERCIST, and PERCIMT well-differentiated those with longer PFS (*p* < 0.001, *p* = 0.009) and OS (*p* = 0.001, *p* = 0.002). In the multivariate analysis, performance status [hazard ratio (HR) = 0.278, *p* = 0.015], imRECIST (HR = 3.799, *p* = 0.026), and imPERCIST (HR = 4.064, *p* = 0.014) were predictive factors for PFS, while only performance status (HR = 0.327, *p* = 0.035) and imPERCIST (HR = 3.247, *p* = 0.007) were predictive for OS.

**Conclusions:** At the first evaluation during treatment with ICI, imPERCIST criteria correctly evaluated treatment response and appeared able to predict survival. Moreover, in patients with iSD on CT, imPERCIST were able to discriminate those with longer survival. This advantage might allow for earlier therapy modification based on metabolic response.

## Introduction

Several clinical studies have demonstrated the successful therapeutic approach of immune checkpoint inhibitors (ICIs) in patients affected by different malignancies when compared with chemotherapy. As a matter of fact, these new agents, acting against cytotoxic T-lymphocyte-associated antigen (CTLA)-4, and anti-programmed death (PD)-1 or its ligand (PD-L1), have been approved so far for over 18 types of cancer (1–3). However, owing to the peculiar response patterns observed in these immune-modulating agents, in parallel with the increased use of ICI, also the assessment of tumor response by medical imaging has become more challenging. Indeed, PD-1/L1 and CTLA-4 blockage aims to restore the immune response by recalling neutrophils, macrophages, and activating T cells within the tumor microenvironment. Consequently, because of tumor inflammation, malignant lesions might appear stable, or even larger either in size or in metabolic activity before effective shrinkage occurs, making it difficult to discriminate between true progression from the so-called pseudo-progression (4–7). To overcome these limitations, numerous response criteria have been proposed, starting with the traditional Response Evaluation Criteria in Solid Tumors (RECIST) 1.1 (8). The main peculiarity of the new morphological criteria developed in the ICI era, such as immune-related response criteria (irRC) and immune-modified (im)RECIST, is that the appearance of new lesions is not always synonymous with progression of disease, but requires confirmation at least after 4–8 weeks (9, 10). Likewise, metabolic criteria based on ^18^F-fluorodeoxyglucose positron emission tomography/computed tomography (^18^F-FDG PET/CT) have been modified aiming to improve diagnostic accuracy during immunotherapy. Of note, new lesions are considered a sign of progression according to their number and size or if metabolic activity is greater than a determined cut-off, as proposed by PET Response Evaluation Criteria for Immunotherapy (PERCIMT) and Immunotherapy-modified PET Response Criteria in Solid Tumors (imPERCIST) criteria, respectively (11, 12).

The purpose of the present study was to investigate the concordance between morphological and metabolic criteria for early response evaluation and to correlate findings with survival in patients with advanced non-small cell lung cancer (NSCLC) undergoing treatment with checkpoint inhibitors.

## Materials and Methods

### Study Population

**Figure 1:**
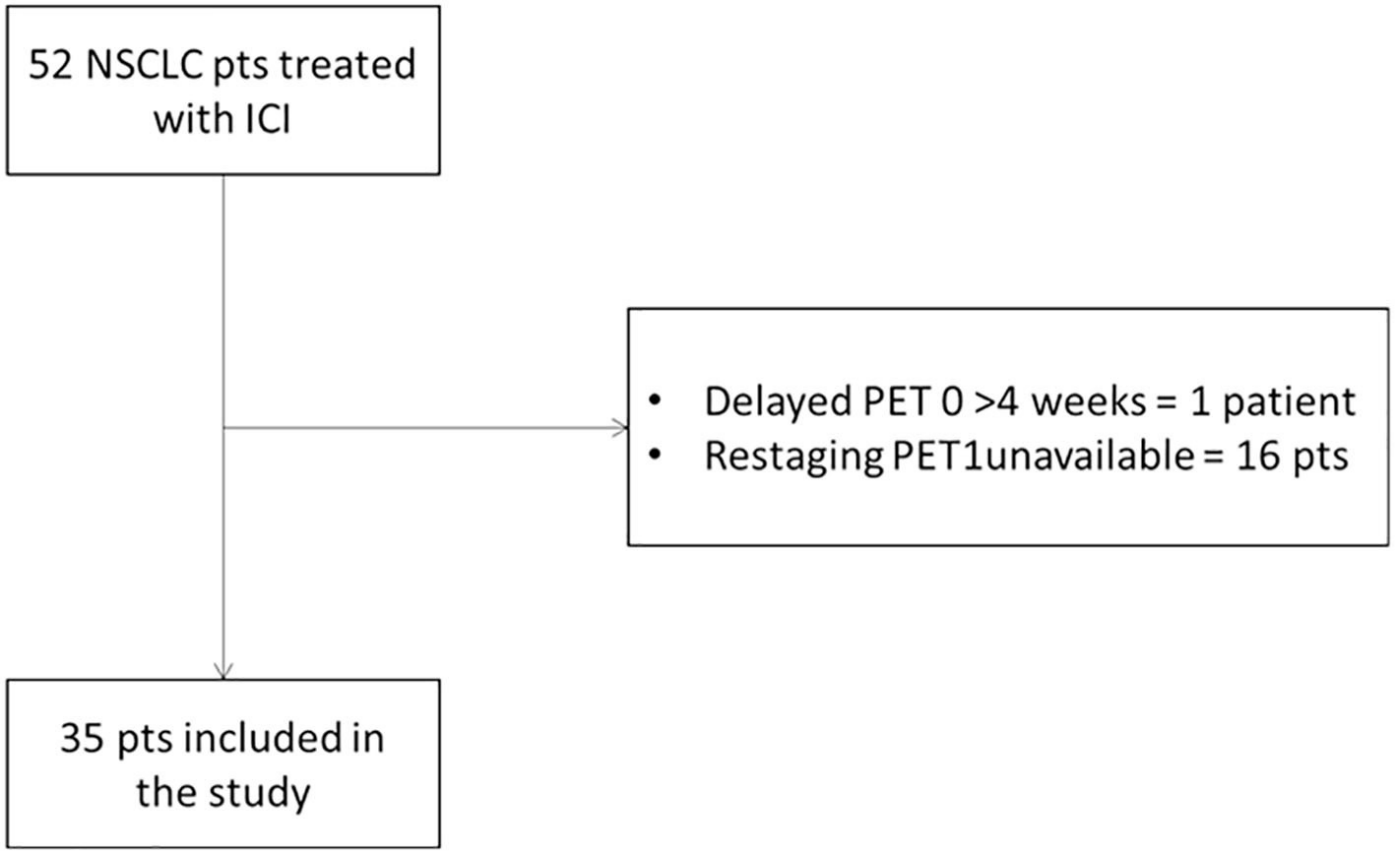
Flowchart of patient accrual.

From December 2015 to May 2019, patients with histopathologically proven advanced NSCLC who were scheduled to undergo ICI treatment were enrolled. Prospective data were collected from patients (*n* = 42) adhering to the same diagnostic trial, registered at https://clinicaltrials.gov/ (NCT03563482), and from other clinical trials for ICI (*n* = 10). Eligible patients were required to have both contrast-enhanced CT and ^18^F-FDG PET/CT scan within 1 month before starting ICI and a second scan at the first restaging after 3 cycles for pembrolizumab or 4 cycles for nivolumab (Figure 1). Moreover, all patients repeated CT every 3 or 4 cycles until confirmed progression. Other exclusion criteria were as follows: primary malignancy other than NSCLC; no lesion on ^18^F-FDG PET/CT above the minimum standardized uptake value (SUV) normalized to lean body mass (SUL) as defined by PERCIST (1.5 × liver SUL + 2 SDs of liver SUL) (13); plasma glucose level was ≥200 mg/dL before ^18^F-FDG PET/CT. The study has been approved by the local institutional review board and in accordance with Declaration of Helsinki and Good Clinical Practice guidelines. Written informed consent was obtained in all cases.

### Imaging Protocol

#### ^18^F-FDG PET/CT

PET/CT scans were performed as previously described (14). A GE ADW4.6 workstation (GE Healthcare, Waukesha, WI, USA) was used to display images, which were interpreted by two experienced nuclear medicine physicians. For the semi-quantitative analysis, the threshold of the volumes of interest (VOIs) was set at 0.5 by PETVCAR (GE Healthcare). The maximum SUV (SUVmax) was defined as the value of the highest pixel and average SUV (SUVmean) as the mean SUV related to the tumor burden. To determine the peak SUV corrected for lean body mass (SULpeak), the reviewer placed a sphere or cube as the VOI around the hottest lesions (up to five lesions, no more than two per organ). Within this VOI, the software searched for the 1.0-cm^3^ sphere that encompassed the voxels with the highest average SUL. For background activity, a 3-cm-diameter spherical VOI was delineated in the right lobe of the liver or in the descending thoracic aorta for patients with liver involvement. Response of SULpeak (%) was defined as (sum of baseline SULpeak—sum of follow-up SULpeak)/(sum of baseline SULpeak) × 100. Target lesions on follow-up scans were not necessarily the same as target lesions at baseline (13).

### Response Assessment

Two physicians (E.L., A.C.), specializing in immunotherapy evaluation, reviewed all consecutive scans to reach a consensus. Morphological evaluation was determined according to RECIST 1.1 and imRECIST (8, 10). Metabolic response on ^18^F-FDG PET/CT was defined according to the European Organization for Research and Treatment of Cancer (EORTC) criteria, PERCIST, and its variation imPERCIST (12, 13, 15). Response Evaluation Criteria for Immunotherapy (PERCIMT) were also considered in our analysis (11). Supplementary Table 1 details the response categories. Briefly, we consider four response categories: complete response (CR), partial response (PR), stable disease (SD), and progressive disease (PD) for morphological criteria. Likewise, complete metabolic response (CMR), partial metabolic response (PMR), stable metabolic disease (SMD), and progressive metabolic disease (PMD) were considered for metabolic criteria.

### Statistical Analysis

The concordance among response criteria was assessed using Cohen's κ coefficient. Agreement between the two assessments was categorized as poor (weighted κ < 0.2), fair (weighted κ = 0.21–0.40), moderate (weighted κ = 0.41–0.60), good (weighted κ = 0.61–0.80), and almost perfect (weighted κ > 0.80) (16). Progression-free survival (PFS) was calculated as the interval from the date of initiation of ICI to the date of either disease progression or death, whereas overall survival (OS) was calculated as the duration between the date of initiation of immunotherapy and the date of death from any cause (17). PFS and OS were analyzed using the Kaplan–Meier method and log-rank test. Then, forward stepwise multivariate regression analysis was performed to identify factors correlated with PFS and OS based on the calculation of hazard ratios (HRs) and 95% CI (14). Variables included in the final multivariate analysis were selected according to their clinical relevance and statistical significance in a univariate model (cut-off, *p* < 0.10). All statistical analyses were carried out using the Statistical Package for Social Sciences, version 23.0, for Windows (SPSS, Chicago, IL), and *p* < 0.05 were considered to be statistically significant (17).

## Results

### Patient Characteristics

Out of the 52 patients with metastatic NSCLC enrolled in the clinical trial, 35 patients (23 men and 12 women) were included in the analysis as they had both CT and ^18^F-FDG PET/CT at baseline and at the first restaging. Patients were treated with a standard schedule of nivolumab (*n* = 19), pembrolizumab (*n* = 14), and nivolumab/ipilimumab (*n* = 2). Twelve patients (34.3%) presented at diagnosis with advanced metastatic NSCLC, whereas the other 23 patients (65.7%) were treated with one or more anticancer therapies. The median number of immunotherapy cycles was 9 (range, 2–47).

The clinical characteristics of patients are summarized in Table 1.

**Table 1:** Patient characteristics.

	***N* (%)**
**Age** median (range)	75 (51–86)
**Gender**	
Male	23 (65.7)
Female	12 (34.3)
**Smoking history**	
Former/current	31 (88.6)
Never	4 (11.4)
**Performance status**	
0	19 (54.3)
≥1	16 (45.7)
**Line of treatment**	
0	12 (34.3)
1	12 (34.3)
≥2	11 (31.4)
**Histology**	
Adenocarcinoma	25 (71.4)
Squamous cell carcinoma	6 (17.1)
Other	4 (11.5)
**Tumor PD-L1 expression level**	
Positive	15 (42.9)
Negative	8 (22.9)
Indeterminate or missing	12 (34.2)

### Response Comparison for imRECIST and Standard Metabolic Criteria (EORTC, PERCIST)

In our study, all cases of PD at first evaluation according to RECIST 1.1 were all confirmed after at least 4 weeks according to imRECIST. As response rates between RECIST 1.1 and imRECIST were comparable, we used only the latter for our analysis. Classification between imRECIST and EORTC criteria was concordant in 20 patients (57.1%) with a moderate agreement between the two assessments (κ = 0.340, Table 2A). In particular, the change of response category was most frequently seen in patients classified as SD by imRECIST criteria: of 15 patients with SD, 6 (40%) were reclassified to CMR/PMR as the decrease in the sum of the diameters of the target lesions was <30%, while the decrease in the sum of SUVmax was more than 25%, whereas another 6 (40%) were reclassified to PMD by EORTC as new lesions were detected on ^18^F-FDG PET/CT. Similar levels of agreement were obtained comparing imRECIST and PERCIST criteria (κ = 0.342, Table 2B).

**TABLE 2A:** Comparison between imRECIST and metabolic criteria (EORTC).

**imRECIST**	**EORTC**				
	**CMR**	**PMR**	**SMD**	**PMD**	**Total**
CR	0	0	0	0	0
PR	1	2	0	1	4
SD	1	5	3	6	15
PD	0	1	0	15	16
Total	2	8	3	22	35

**TABLE 2B:** Comparison between imRECIST and metabolic criteria (PERCIST).

**imRECIST**	**PERCIST**				
	**CMR**	**PMR**	**SMD**	**PMD**	**Total**
CR	0	0	0	0	0
PR	1	2	0	1	4
SD	1	5	4	5	15
PD	0	1	1	14	16
Total	2	8	5	20	35

*CR, complete response; PR, partial response; SD, stable disease; PD, progressive disease; CMR, complete metabolic response; PMR, partial metabolic response; SMD, stable metabolic disease; PMD, progressive metabolic disease*.

### Response Comparison for imRECIST and Immune-Related Metabolic Criteria (imPERCIST, PERCIMT)

imRECIST and imPERCIST were discordant in 20 patients (57.1%) with low agreement in the response classification between the two assessments (κ = 0.143, Table 3A). When adopting imPERCIST criteria, tumor responses were upgraded in 2 (10%) patients and downgraded in 18 (90%) patients. Notably, of 16 patients classified as PD according to imRECIST, 9 were reclassified as SMD according to imPERCIST, as the increase in the sum of the longest diameters of the target lesions was more than 20%, while the increase of overall SULpeak was <30%, and 2 patients as PMR because SULpeak reduction was >30%. Furthermore, of 15 patients classified as SD according to imRECIST, 1 was classified as PMD according to imPERCIST, as new lesions were detected on PET/CT contributing to summed SULpeak for PMD, but not on CT, and 6 patients as PMR, as the decrease in the sum of the longest diameters of the target lesions was <30%, while the decrease in the SULpeak was more than 30%. On the other hand, the level of agreement was higher, although moderate, when comparing imRECIST with PERCIMT (κ = 0.413), with concordance in 23 patients (65.7%). Overall, 9 patients were downgraded and 3 upgraded (Table 3B).

**TABLE 3A:** Comparison between imRECIST and immuno-related metabolic criteria (imPERCIST).

**imRECIST**	**imPERCIST**				
	**CMR**	**PMR**	**SMD**	**PMD**	**Total**
CR	0	0	0	0	0
PR	1	2	0	1	4
SD	1	5	8	1	15
PD	0	2	9	5	16
Total	2	9	17	7	35

**TABLE 3B:** Comparison between imRECIST and immuno-related metabolic criteria (PERCIMT).

**imRECIST**	**PERCIMT**			
	**CMR**	**SMD**	**PMD**	**Total**
CR	0	0	0	0
PR	1	3	0	4
SD	1	11	3	15
PD	0	4	12	16
Total	2	18	15	35

*CR, complete response; PR, partial response; SD, stable disease; PD, progressive disease; CMR, complete metabolic response; PMR, partial metabolic response; SMD, stable metabolic disease; PMD, progressive metabolic disease*.

### Clinical Outcome and Prognosis

**Figure 2:**
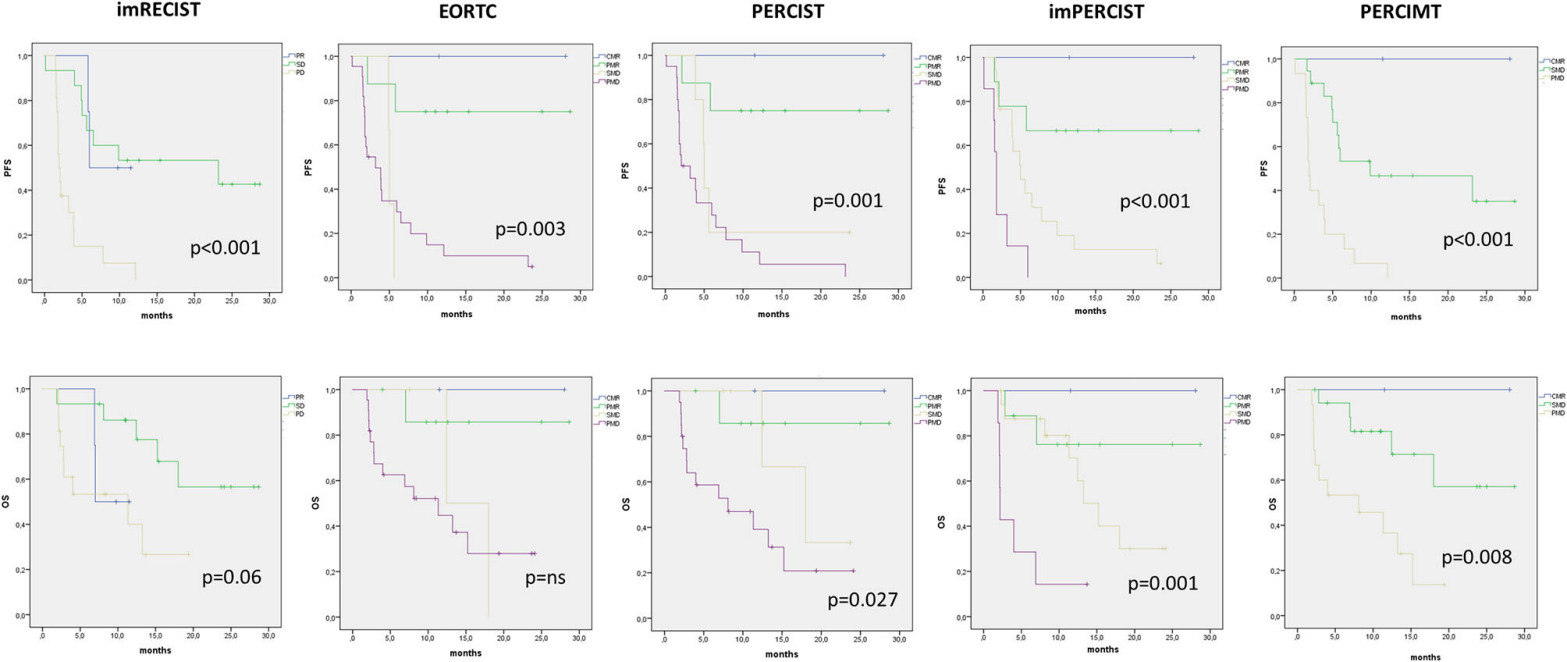
Kaplan–Meier curves with log-rank (Mantel–Cox) test obtained for PFS and OS according to the different morphological and metabolic response criteria.

**Figure 3:**
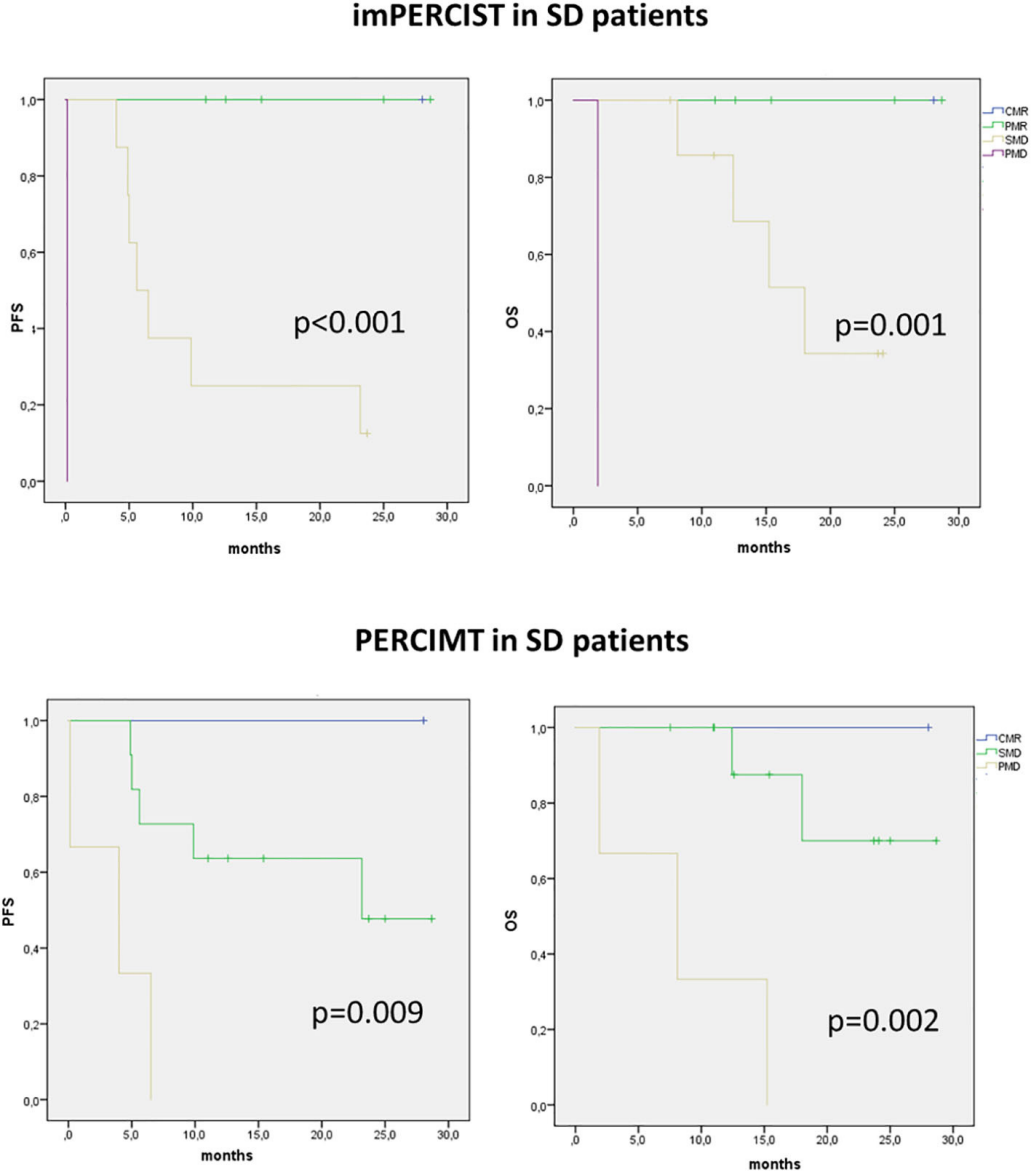
Kaplan–Meier curves with log-rank (Mantel–Cox) test obtained for PFS and OS in patients presenting with SD on imRECIST and classified according to immune-related metabolic criteria (imPERCIST, PERCIMT).

The median duration of follow-up was 13.7 months (range, 2–28.7 months). Median PFS and OS for all patients was 5.6 months (95% CI, 3.1–8.1 months) and 15.3 months (95% CI, 8.9–21.6 months), respectively. With imRECIST, the median PFS was 6 months for patients with PR, 23 months in those with SD, and 2 months in those with PD. The median PFS in patients with PR was significantly longer than in those with PD (*p* = 0.031), but was not significantly longer than in those with SD (Figure 2). Among all metabolic parameters, PMD rate was comparable according EORTC, PERCIST, imPERCIST, and PERCIMT, with a median PFS of 3.2, 2.6, 1.8, and 1.9 months, respectively (Figure 2). However, while there was no statistical difference between SMD and PMD according to EORTC and PERCIST criteria, patients with SMD according imPERCIST and PERCIMT had longer PFS than those with PMD (*p* = 0.004 and *p* < 0.001, respectively). At the time of analysis, 16 patients (45.7%) had died. OS curve according to EORTC criteria was not significant (Figure 3) and showed only a tendency for imRECIST criteria (*p* = 0.06) (Figure 2). On the other hand, PERCIST, imPERCIST, and PERCIMT were significantly associated with OS (*p* = 0.027, *p* = 0.001, and *p* = 0.008, respectively), with similar survival for CMR/PMR group (median not reached) (Figure 2). Moreover, OS between SMD and PMD according to imPERCIST and PERCIMT was statistically significant (*p* = 0.002 and *p* = 0.006, respectively), whereas according to PERCIST, it was not.

We then analyzed the value of immune-metabolic criteria, i.e., imPERCIST and PERCIMT, in patients showing SD on CT. In these patients, both imPERCIST and PERCIMT well-differentiate patients with longer survival, expressed by both PFS (*p* < 0.001 and *p* = 0.009, respectively) and OS (*p* < 0.001 and *p* = 0.002, respectively) (Figure 3).

Finally, we also performed a multivariate analysis including all clinical variables and response criteria which were significant at univariate Cox proportional-hazards model. According to our results, performance status (HR = 0.278, *p* = 0.015), imRECIST (HR = 3.799, *p* = 0.026), and imPERCIST (HR = 4.064, *p* = 0.014) were predictive factors for PFS, while only performance status (HR = 0.327, *p* = 0.035) and imPERCIST (HR = 3.247, *p* = 0.007) were predictive for OS (Table 4).

**TABLE 4:** Univariate and multivariate Cox proportional-hazards regression analysis for prediction of PFS and OS.

**Parameters**	**PFS**			**OS**		
	**Hazard ratio**	**95% CI**	***P*-value**	**Hazard ratio**	**95% CI**	***P*-value**
Age (median)	0.935	0.423–2.067	ns	1.110	0.413–2.984	ns
Gender	0.519	0.231–1.164	ns	0.332	0.122–0.905	**0.031**
Smoking history	1.407	0.480–4.129	ns	2.668	0.747–9.545	ns
Histology	0.700	0.208–2.356	ns	1.701	0.583–4.962	ns
Performance status	0.479	0.216–0.998	**0.071**	0.289	0.102–0.815	**0.019**
imRECIST	3.962	1.826–8.596	**0.001**	1.893	0.786–4.560	ns
EORTC	2.330	1.360–3.994	**0.002**	2.445	1.116–5.359	**0.026**
PERCIST	2.572	1.483–4.460	**0.001**	3.020	1.289–7.078	**0.011**
imPERCIST	3.388	1.826–8.596	**0.001**	3.904	1.701–8.958	**0.001**
PERCIMT	3.321	1.571–7.019	**0.002**	4.157	1.477–11.706	**0.007**
**Multivariate cox proportional-hazards regression analysis**						
Gender	–	–	–	0.729	0.301–1.524	ns
Performance status	0.278	0.099–0.791	**0.015**	0.327	0.116–0.922	**0.035**
imRECIST	3.799	1.169–12.340	**0.026**	–	–	–
EORTC	0.730	0.078–6.800	ns	0.005	0.001–2.394	ns
PERCIST	0.890	0.086–9.256	ns	1.235	0.053–2.210	ns
imPERCIST	4.064	1.329–12.426	**0.014**	3.247	1.385–7.611	**0.007**
PERCIMT	1.742	0.418–7.258	ns	3.749	0.635–22.114	ns

*ns, not significant; OS, overall survival; PFS, progression-free survival*.

## Discussion

Immunotherapy with ICI has introduced new challenges for medical imaging. This involves anatomical imaging, such as CT or MRI, as well as functional imaging, expressed by nuclear medicine techniques. In fact, with the growing use of ICI, atypical response patterns have been detected and described, such as pseudo-progression, hyper-progression, and dissociated response (14, 18). With this regard, one of the primary goals for the medical community is the early identification of patients who will not respond to ICI to permit a rapid switch of therapeutic line, to reduce the risk of immune-related adverse events, and to decrease the economic impact of these drugs, which remain very expensive. At the same time, it is important to avoid the premature treatment withdrawal for patients with therapeutic benefit (19, 20). For these reasons, many different immune-related scales have been proposed in the last years, but none of them has been routinely adopted in clinical practice, hence the debate is still open (21). Furthermore, only few studies have been published providing a direct comparison between CT-based and PET-based criteria, most with small cohorts and in melanoma setting (22–25). Only Rossi et al. (26) have recently compared anatomic and metabolic criteria in NSCLC patients treated with nivolumab.

In our study, we aimed to compare different response criteria in patients with advanced NSCLC treated with anti-PD-1/PD-L1 at first evaluation, after approximately 8 weeks. We adopted standard anatomic criteria as RECIST 1.1 and its immune-variation imRECIST, the latter combining cut-off values and unidimensional size of RECIST 1.1 and irRC criteria for interpretation of new lesions. Indeed, the main caveat is that irRC criteria require bidimensional measurements of tumor lesions hardly to apply in routine. Moreover, along with standard EORTC and PERCIST criteria, we also investigated imPERCIST and PERCIMT criteria. The latter can be considered the variation of irRC in which the metabolic dimensions of new lesions are embedded in the overall tumor burden (11).

In our study, we demonstrated a low overall agreement between imRECIST and imPERCIST, particularly for patients in the PD category. In fact, more than half of patients, i.e., 69%, classified as PD were downgraded to either SMD (9/16) or PMR (2/16) according to imPERCIST. Our results suggest that the sum of SULpeak from new lesions appears more reliable than diameter measurement, allowing to detect a therapeutic response as early as 8 weeks since ICI started. Hence, imPERCIST could help to avoid an early interruption of ICI therapy. On the other hand, our findings showed a moderate agreement between imRECIST and PERCIMT, with only 4 out of 16 patients with PD downgraded according to SMD, highlighting once again that metabolic activity expressed by SULpeak is optimal than metabolic measurement for new lesions. This is in line with a recent study in melanoma patients treated with ICI, where PERCIMT criteria were demonstrated suboptimal for the identification of disease progression (22). Furthermore, our results are apparently opposite to those of Rossi et al. (26), who compared different PET- and CT-based response criteria in a similar cohort. In fact, they demonstrated limited prognostic value of the SMD group who had a survival similar to PMD patients. However, in their study, no significant difference between PERCIST and imPERCIST was found, whereas in our study among 20 patients classified as PMD according PERCIST, 13 were downgraded to SMD or PMR. In our opinion, this is the main reason for the different results obtained between our study and that from Rossi et al.

As shown in the Kaplan–Meier curves, we observed a positive impact of early PET and CT response on PFS, while only metabolic immune-related response criteria were prognostic for OS, confirming the role of ^18^F-FDG PET/CT in predicting final clinical response to immunotherapy already underscored in previous studies in melanoma (24, 25). In fact, as visible on imRECIST survival curves, SD patients had a survival profile similar to PR curve. When selecting only patients with SD by imRECIST, both imPERCIST and PERCIMT criteria were able to identify three further survival curves (Figure 3). This evidence supports the hypothesis that SD group comprises a heterogeneous cohort with different prognosis, some with clinical benefit and others without. Hence, from this perspective, immune-related response criteria could be useful for monitoring the efficacy of immunotherapy, by identifying responders vs. non-responders as well as by predicting clinical outcomes, as arisen from our multivariate analysis.

Nevertheless, our study presents some limitations. The main one is related to its relatively small cohort. Furthermore, the use of different PET/CT scanners may have caused some variability in metabolic parameter measurement, although all patients were imaged in the same scans throughout the study. Third, in our study we have investigated the most validated ^18^F-FDG PET-based criteria so far, whereas we did not consider other metabolic parameters or variables, such as metabolic tumor volume, total lesion glycolysis, circulating tumor cells, neutrophil-to-lymphocyte ratio, and their combination, which recently have been demonstrated to predict PFS and OS in patients treated with ICI (17, 27, 28).

In conclusion, our study encourages the use of immune-metabolic response criteria by ^18^F-FDG PET/CT, in particular imPERCIST, to assess early response and to predict long-term outcomes in patients with NSCLC under ICI therapy. However, our first findings need to be validated in a larger prospective study.

## Data Availability Statement

The raw data supporting the conclusions of this article are available from the corresponding author, upon reasonable request.

## Ethics Statement

The studies involving human participants were reviewed and approved by Comitato Etico Humanitas. The patients/participants provided their written informed consent to participate in this study.

## Author Contributions

AC: protocol development, data collection and management, data analysis, and manuscript writing. EL: project development, data collection and management, and manuscript editing. SR and LT: protocol development and data collection and management. All authors: contributed to the article and approved the submitted version.

## Conflict of Interest

The authors declare that the research was conducted in the absence of any commercial or financial relationships that could be construed as a potential conflict of interest.
