# Supplementary material for: Comparison of Metabolic and Morphological Response Criteria for Early Prediction of Response and Survival in NSCLC Patients Treated With Anti-PD-1/PD-L1
Source: Front Oncol. 2020 Jul 31;10:1090. doi: 10.3389/fonc.2020.01090 (PMC7412129; doi:10.3389/fonc.2020.01090)
Supplement: Supplementary file 1 [file Table_1.doc]

**Supplemental Table 1.** Summary of anatomic and metabolic response criteria adopted in the study.

|  | **RECIST 1.1** | **imRECIST** | **PERCIST** | **imPERCIST** | **PERCIMT** |
| --- | --- | --- | --- | --- | --- |
| **CR/CMR** | disappearance of all lesions | as RECIST 1.1 | Disappearance of all metabolically active lesions | same as PERCIST | Disappearance of all metabolically active lesions  no new lesions |
| **PR/PMR** | ≥30% decrease from baseline | as RECIST 1.1 | SULpeak reduction ≥30% in the hottest target lesions | same as PERCIST | Decrease of preexisting metabolic lesions  no new lesions |
| **SD/SMD** | Neither PD nor PR/CR | Neither PD nor PR/CR | Neither PMD nor PMR/CMR | Neither PMD nor PMR/CMR | Neither PMD nor PMR/CMR |
| **PD/PMD** | ≥20% increase, minimum 5mm | as RECIST 1.1 | SULpeak increase ≥30% in the hottest target lesion | SULpeak increase ≥30% in the hottest target lesion | 4 or more new lesions (<1cm in diameter), or  3 or more new lesions (>1cm in diameter), or  2 or more new lesions (>1.5cm in diameter) |
| **new lesions** | unequivocal PD | Incorporated in the sum of measurements | unequivocal PMD | do not configure automatically PMD, incorporate in the sum of SULpeak | according to the number and the diameter |
